# Inactivation of aPKCλ Reveals a Context Dependent Allocation of Cell Lineages in Preimplantation Mouse Embryos

**DOI:** 10.1371/journal.pone.0007117

**Published:** 2009-09-21

**Authors:** Nicolas Dard, Tran Le, Bernard Maro, Sophie Louvet-Vallée

**Affiliations:** 1 CNRS, UMR7622 - Laboratoire de Biologie Cellulaire du Développement, 9 Quai Saint-Bernard, Bâtiment C, Paris, France; 2 UPMC Univ. Paris 06, UMR7622 - Laboratoire de Biologie Cellulaire du Développement, 9 Quai Saint-Bernard, Bâtiment C, Paris, France; 3 Sackler Faculty of Medicine, Tel Aviv University, Ramat Aviv, Israel; University of Birmingham, United Kingdom

## Abstract

**Background:**

During mammalian preimplantation development, lineage divergence seems to be controlled by the interplay between asymmetric cell division (once cells are polarized) and positional information. In the mouse embryo, two distinct cell populations are first observed at the 16-cell stage and can be distinguished by both their position (outside or inside) and their phenotype (polarized or non-polarized). Many efforts have been made during the last decade to characterize the molecular mechanisms driving lineage divergence.

**Methodology/Principal Findings:**

In order to evaluate the importance of cell polarity in the determination of cell fate we have disturbed the activity of the apical complex aPKC/PAR6 using siRNA to down-regulate aPKCλ expression. Here we show that depletion of aPKCλ results in an absence of tight junctions and in severe polarity defects at the 16-cell stage. Importantly, we found that, in absence of aPKCλ, cell fate depends on the cellular context: depletion of aPKCλ in all cells results in a strong reduction of inner cells at the 16-cell stage, while inhibition of aPKCλ in only half of the embryo biases the progeny of aPKCλ defective blastomeres towards the inner cell mass. Finally, our study points to a role of cell shape in controlling cell position and thus lineage allocation.

**Conclusion:**

Our data show that aPKCλ is dispensable for the establishment of polarity at the 8-cell stage but is essential for the stabilization of cell polarity at the 16-cell stage and for cell positioning. Moreover, this study reveals that in addition to positional information and asymmetric cell divisions, cell shape plays an important role for the control of lineage divergence during mouse preimplantation development. Cell shape is able to influence both the type of division (symmetric or asymmetric) and the position of the blastomeres within the embryo.

## Introduction

During development two major mechanisms are involved to generate cell diversity: asymmetric cell divisions, leading to the formation of two different daughter cells, and cellular interactions, leading previously identical cells to adopt distinct fate. The morphogenesis of the mouse blastocyst provides an excellent system to study these two mechanisms. During preimplantation development, two distinct cell populations are first observed at the 16-cell stage that can be distinguished by both their position (outside and inside) and their phenotype (polarized and non-polarized, respectively). This difference is maintained in the blastocyst where a layer of epithelial cells, the trophectoderm, surrounds a cavity and a group of undifferentiated cells, the inner cell mass.

In the late 60s, it was shown that changing the position of blastomeres within the embryo could change their fate [1]. This observation led Tarkowski and Wroblewska to propose the « inside - outside » model where the position of a blastomere at the 16-cell stage drives it towards a trophectodermal (outside) or an inner cell mass (inside) fate [2]. In outer cells, the area of the membrane facing the external milieu is not engaged in cell-cell contacts (asymmetry of contacts), while inner cells are completely surrounded by other cells. This leads outside cells to differentiate into epithelial cells while inner cells remain undifferentiated. These observations emphasize the role of extrinsic factors (cell interactions) during blastocyst morphogenesis.

In the early 80s, a cell biological approach led Johnson and colleagues to propose that the phenotypic divergence between inside cells and outside cells resulted from the polarization of blastomeres at the 8-cell stage during compaction and from its unequal inheritance through asymmetric divisions at the 8- to 16-cell stage transition [3]. At the 8-cell stage, during compaction, each blastomere polarizes its cytoplasm and its cortex along an apico-basal axis, and the development of an adhesive basolateral cortical domain leads to the flattening of blastomeres upon one another (for review, see [4]). Cell polarization at the 8-cell stage depends on extrinsic (cell adhesion, basolateral cue) and intrinsic (microtubule network, apical cue) factors [5]. During the following mitosis, cytoplasmic polarity is lost, but the apical cortical domain remains stable, allowing blastomeres that inherit it to re-establish cell polarity. Moreover, since the axis of polarity at the 8-cell stage is radial, after an asymmetric cell division the polarized daughter cell is already located at the periphery while the non-polarized cell is positioned towards the inside of the embryo. At the 16-cell stage, because the apical surface is less adhesive than the basolateral membrane, polar cells tend to engulf non-polar cells that are uniformly adhesive, thus reinforcing the positioning of these polarized cells at the periphery of the embryo [6].

Another mechanism plays an important role during blastocyst morphogenesis: cell shape. Cell shape is dependent upon different factors such as cortical tension, adhesive properties and the cellular environment. Cell shape plays a major role to control the number of inside and outside cells during the 16- to 32-cell stage transition [7]–[9]. The number of polarized blastomeres that divide asymmetrically is very variable during the 8- to 16-cell stage transition, because spindle orientation is not controlled [10]. Therefore in the 16-cell stage embryo, the outer/inner cell ratio in most embryos lies in the range between 9/7 and 14/2 [9]. However, this ratio is much less variable at the 32-cell. Indeed, the number of inner and outer cells influences cell shape at the 16-cell stage, and consequently spindle orientation during the 16- to 32-cell stage transition: when there is a low number of inner cells, outside cells are elongated along the radial axis of the embryo and mitotic spindles adjust along this radial axis and thus outer cells tend to divide asymmetrically, thereby increasing the number of inner cells. Reversely, when the number of inside cells is high, outer cells are flattened at the surface of the embryo and mitotic spindles orientate parallel to the surface, and thus outer cells tend to divide symmetrically, thereby maintaining the inner/outer cell ratio (dividing inner cells always give rise to two inner daughter cells).

Many efforts have been made to characterize the molecular mechanisms driving blastocysts morphogenesis, and especially inner cell mass and trophectodermal cells allocation, during the last decade. In particular, the maintenance of inner cell mass (ICM) and trophectoderm (TE) fates requires the transcription factors Cdx2 and Oct4, whose expression is restricted to the TE and ICM, respectively, after the initiation of blastocyst formation [11]–[13]. Cdx2 is required for the repression of *oct4* in the TE. However, this protein is dispensable for TE specification since *cdx2* -/- embryos form expanded blastocysts. The correlation between *cdx2* expression and lineage allocation is controversial. Actually, a position independent variability of *cdx2* expression is observed from the 16-cell to 32-cell stage [14], [15]. Moreover, *cdx2* mutant blastomeres contribute to both ICM and TE and the expression and localization of aPKC is not affected [15]. Whereas these two papers conclude that early stochastic processes control their expression pattern and that the definite pattern is established downstream of lineage allocation, another study claims that the expression level of *cdx2* influences cell position and the overexpression of *cdx2* in blastomeres induced a more extensive and concentrated aPKC localization to the apical poles of the blastomeres [16].

Thus, blastocyst morphogenesis and early lineage divergence seem to be controlled by the interplay between cellular mechanisms involving positional information, cell-cell interactions, cell shape, cell polarization and asymmetric cell divisions. In order to evaluate the importance of cell polarity in the determination of cell fate we have decided to disturb the activity of the aPKC/PAR6 apical complex in different cellular contexts. Indeed, we have previously reported that members of the PAR/aPKC protein family are expressed in the preimplantation mouse embryo and display a polarized distribution from the 8-cell stage onwards [17]. Interestingly, the PAR6/aPKC complex accumulates at the apical cortex at the onset of compaction at the 8-cell stage and remains stable during mitosis at the 8- to 16-cell stage transition [17], [18]. While PAR6 is maternally expressed [17], aPKCλ and aPKCζ are not [17], [18], allowing inactivation of their activity by RNA interference. Down-regulation of aPKC in a small number of blastomeres was previously reported to increase the number of inner cells through a modification of spindle orientation [19]. Here, we show that when early stabilization of cell polarity is impaired by inactivation of aPKCλ in all or half blastomeres , cell allocation is dependent upon the cellular context and controlled by cell shape and is independent of spindle orientation.

## Materials and Methods

### Ethic Statement

All animals used in experiments reported in this publication were housed and handled by persons skilled by institutional committee according to CNRS and French Agriculture Department (agreement #A75-05-13).

### Recovery and culture of mouse embryos

Recovery and culture of embryos were performed as described previously [20]. Briefly, 9 to 12 weeks old females OF1 (Charles River) were super-ovulated by intra-peritoneal injection of 5 UI Pregnant Mare Serum gonadotrophin (PMS, Intervet) and 5 UI human Chorionic Gonadotrophin (hCG, Intervet), 48 hours later. Females were mated with OF1 males (fertilization occurs about 12 hours post-hCG). Two-cell stage embryos were collected by flushing oviducts in M2+BSA (4 mg/ml) medium and then cultured in T6+BSA under paraffin oil at 38°C in 5% CO_2_.

### Antibodies and reagents

TRITC-conjugated phalloïdin was purchased from Sigma. For immunofluorescence, rabbit anti-ezrin (provided by P. Mangeat), mouse anti-aPKCλ (BD Bioscience), rabbit anti-aPKCζ (clone C-20; Santa Cruz), ECCD2 rat anti-E-cadherin (Zymed), mouse anti-ZO1 (Zymed), rabbit anti-cingulin (Zymed), rabbit anti-phospho-myosin light chain 2 (Cell signaling), rabbit anti-megalin [21] antibodies were used at the following dilutions 1∶400, 1∶150, 1∶100, 1∶200, 1∶200, 1∶1000, 1∶250, 1∶500, respectively. Secondary alexa fluor 568 anti-rabbit IgG, alexa fluor 568 anti-mouse IgG, and alexa fluor 488 anti-rat IgG antibodies (Invitrogen) were used at the dilution 1∶600.

### Fixation and staining of embryos

For aPKCλ, aPKCζ, ezrin, E-cadherin, ZO1, and megalin staining, samples were fixed in 3.7% formaldehyde (BDH) in PBS for 30 minutes at 37°C, and neutralized with 50 mM NH_4_Cl in PBS for 10 minutes. Samples were then post-permeabilized with 0.25% Triton X-100 in PBS for 10 minutes, except for aPKCλ samples which were post-permeabilized with 1% SDS in PBS for 20 minutes. For cingulin staining, samples were pre-fixed in 1% formaldehyde for 1 minute at 37°C then fixed in methanol at −20°C for 6 minutes. Actin staining was performed by a 15 minutes incubation of embryos with 1 µg/mL TRITC-conjugated phalloïdin at room temperature. Hoescht was used to stain chromatin. For immuno-stainings, antibodies were diluted in PBS/Tween/BSA (PBS containing 0.1% Tween-20 and 3% BSA). Primary antibodies were incubated overnight at 4°C, and secondary antibodies were incubated 1 hour at room temperature. Samples were mounted in citifluor and observed under an inverted microscope equipped with a spinning-disk system or a Leica SP5 confocal microscope (IFR83 imaging facility). For all experiments, both aPKCλ-depleted and control embryos were fixed and stained on the same slides and acquisition parameters were identical.

### Stealth RNAi, plasmids, synthesis of mRNA, and microinjection

Stealth RNAi (MSS207677, MSS207678) designed specifically against aPKCι/λ and stealth RNAi negative control were purchased from Invitrogen, resuspended with DEPC water to prepare 20 µM stock solutions, and injected at a final concentration of 8 µM. For mRNA synthesis, mouse wild type and kinase dead (K273E; gift of T. Hirose) aPKCλ were cloned into pRN3-EGFP-C1 plasmid in BamHI/XhoI sites. Human wild type ezrin was cloned into pRN3-mCherry-N1 plasmid in EcoRI/SalI sites. β5-tubulin-GFP (gift of B. Ludin) was cloned into pRN3 plasmid. In vitro synthesis of mRNA was performed as described previously [22]. Microinjection of stealth RNAi and synthetic mRNAs (10 pl of 0.25 pg/pl) was performed into the cytoplasm of one or two cells of 2-cell stage embryos (35–38 hrs post-fertilization) as described previously [22].

### Time-lapse microscopy

Embryos were cultured in T6+BSA under paraffin oil in a specially designed chamber adapted to the inverted microscope (Axiovert M200, Zeiss), maintained at 38°C, in an atmosphere of 96% air with 4% CO2. The microscope was equipped with a spinning disk (Yokogawa CSU-10) and an EMCCD camera (Hamamatsu). The system was driven by the Volocity Acquisition software (Improvision – Perkin Elmer) running on a Mac Pro (Apple Computer). Series of confocal images (z = 2 µm) were recorded every 15 to 30 min for each channel used (transmission, green and red fluorescence). In these conditions, embryos develop to the blastocyst stage. Statistical analysis was performed using the InStat and Prism software packages (GraphPad).

### Determination of angles in mitotic embryos

Using the Volocity Visualization/Quantitation software package (Improvision – Perkin Elmer) running on a Mac Pro (Apple Computer), the coordinates (x, y, z) of the two poles of the mitotic spindle (in all cells of the embryo) and of the centroid of the embryo were determined. Then, the angle between the vector determined by the two spindle poles and the vector between the centroid of the embryo and the middle of the spindle was calculated using the Numbers software (Apple Computer).

### Measurement of apical and basolateral domains length

16-cell stage embryos stained for actin (TRITC-phalloidin) and chromatin (DAPI) were scanned using a confocal microscope. For each blastomere we selected the slide passing through the nucleus and displaying the largest perimeter in order to measure the length of the apical and basolateral domains using the drawing line tool of the Volocity Visualization/Quantitation software.

### Transmission electron microscopy

Two-cell stage embryos were microinjected in both cells and then cultured in vitro. 16-cell stage embryos were placed in special chambers coated with concanavalin A, fixed in 3% glutaraldehyde in 0,1 M cacodylate buffer (pH = 7,2) and 0,2% tannic acid for 30 min, washed in cacodylate buffer, and postfixed in 0,5% osmium tetroxide in cacodylate buffer for 10 min. Samples were dehydrated in an ethanol series and then stained with uranyl acetate for 10 min. Then, samples were embedded in Epon resin for 72 hours at 60°C, cut on an ultramicrotome and viewed on a Tecnai 12 transmission electron microscope (FEI, Eindoven, The Netherlands) at 80 kV. Digital acquisitions were made with a numeric Keen View camera (Soft and Imaging System).

### Immunoblotting

Embryos were collected in sample buffer, and boiled for 5 min. The total embryo protein content was separated on 8% SDS–polyacrylamide gels and then transferred electrophoretically onto a nitrocellulose membrane. The membranes were blocked in TBS/Tween (150 mM NaCl, 10 mM Tris, pH 7.4, 0.1% Tween 20) containing 3% (w/v) dry milk powder and then incubated with the mouse anti-aPKCλ (dilution 1∶50 in TBS/Tween containing 3% milk) or rabbit anti-aPKCζ antibody (dilution 1∶500) overnight at 4°C. For loading control, membranes were incubated with mouse anti-β−tubulin antibody (Amersham) diluted at 1∶80,000 for 1 hour at room temperature. After washes in TBS/Tween, the membranes were incubated for 1 h at room temperature with anti-mouse (dilution 1∶50,000) or anti-rabbit (dilution 1∶10,000) Ig antibodies linked to peroxidase (Amersham). The membranes were revealed using the Super Signal Western blotting detection system (Pierce) according to the manufacturer's instructions.

## Results and Discussion

### Depletion of aPKCλ induces a destabilization of cell polarity at the 16-cell stage

#### Depletion of aPKCλ in preimplantation mouse embryos

**Figure 1:**
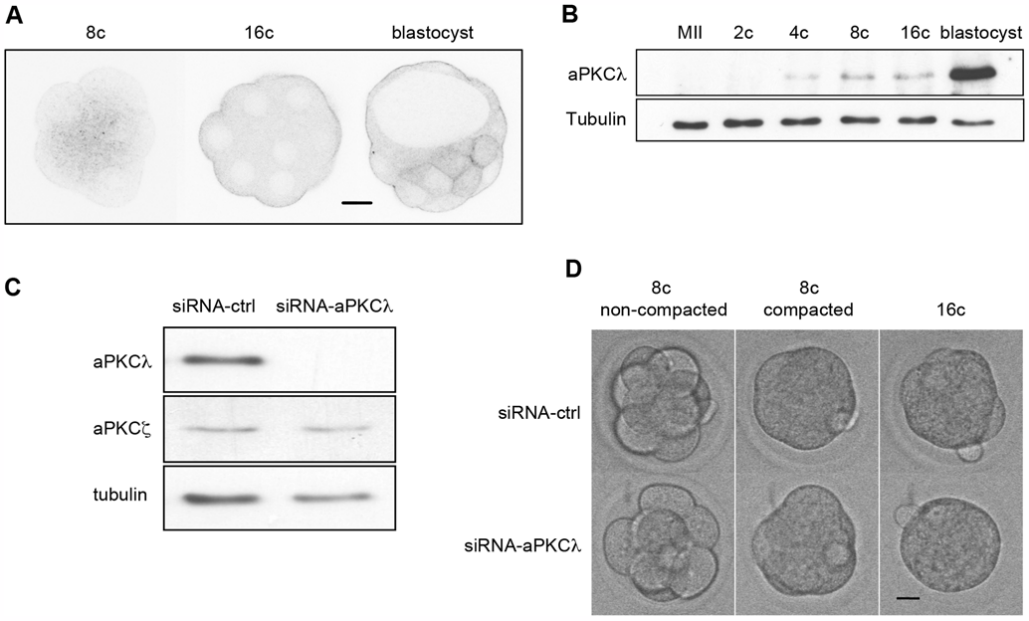
Depletion of aPKCλ induces an abnormal morphology at the 16-cell stage. (A): Spinning-disk microscope analysis of aPKCλ localization at the 8-cell (10 embryos), 16-cell (26 embryos), and blastocyst (13 embryos) stages. Scale bar: 20 µm. (B): Expression profile of aPKCλ protein during preimplantation development. Immunoblot was performed twice on extracts from 50 MII oocytes, 2-cell stage, 4-cell stage, 8-cell stage, 16-cell stage and blastocyst stage embryos. (C): Inhibition of aPKCλ expression by RNA interference. Immunoblot performed on blastocyst extracts. Microinjection of control siRNA or siRNA against aPKCλ was performed at the 2-cell stage in both blastomeres, and embryos were cultured until the blastocyst stage. 40 blastocysts were used per lane. Detection of aPKCζ and tubulin was used as a control for siRNA specificity and a loading control, respectively. Experiments were performed twice with two different siRNA. (D): Snapshots of two living embryos in which control siRNA (siRNA-Ctrl) or siRNA against aPKCλ (siRNA-aPKCλ) were microinjected at the 2-cell stage. Pictures of the same embryo were taken at the non-compacted (8c non-compacted) and compacted (8c compacted) 8-cell stage, and at the 16-cell stage (16c). Scale bar: 20 µm.

aPKCλ was expressed from the 4-cell stage onwards and strongly accumulated in blastocysts (Fig. 1). The protein was detected in the cytoplasm of 8-cell stage blastomeres, and became enriched at the apical cortex of external cells at the 16-cell stage, being predominantly cortical in blastocysts (Fig. 1A). It was also present uniformly at the periphery of inner cells at the blastocyst stage. The expression profile of aPKCζ has already been characterized [17]. Since both proteins are zygotically expressed, we have tried to interfere with their expression by RNA interference. The injection of three different siRNAs against aPKCζ isoform did not efficiently knock-down its expression (data not shown). By contrast, the use of two different siRNAs against aPKCλ resulted in the specific down-regulation of aPKCλ expression as shown by western blot analysis (Fig. 1C). The total depletion of aPKCλ led to embryos displaying a very compacted aspect at the 16-cell stage (Fig. 1D and video S1). Indeed, a careful analysis of embryos shape revealed that the perimeter of the embryos was decreased by about 13% upon aPKCλ down-regulation (Table 1). Moreover, microinjection of mRNA encoding a kinase dead dominant negative mutant of aPKCλ (aPKCλ-K273E) induced the same phenotype (data not shown). In all cases, embryos reached the blastocyst stage and hatched, which is consistent with the finding that aPKCλ knockout embryos can implant, although they die a few days after implantation [23].

**Table 1:** Quantification of the effect of aPKCλ depletion on the shape of the 16-cell stage embryo.

	siRNA-Ctrl (n = 9)	siRNA-aPKCλ (n = 9)
Area (mean±SD)	3403±186 µm^2^	3356±142 µm^2^
Perimeter (mean±SD)	275±30 µm	241±16 µm*
Circularity (mean±SD)	0.71±0.09	0.82±0.05*

*p = 0.034 using the Mann-Whitney test when siRNA-aPKCλ samples were compared to siRNA-Ctrl. The difference was significant.

**p = 0.019 using the Mann-Whitney test when siRNA-aPKCλ samples were compared to siRNA-Ctrl. The difference was significant.

Since it is at the 16-cell stage that the first two cell lineages can be observed for the first time, and because this is dependent upon the setting up of cell polarity and the existence of asymmetric cell divisions, we analyzed this phenotype in more details.

#### Apico-basal polarity is correctly set up at compaction in absence of aPKCλ

**Figure 2:**
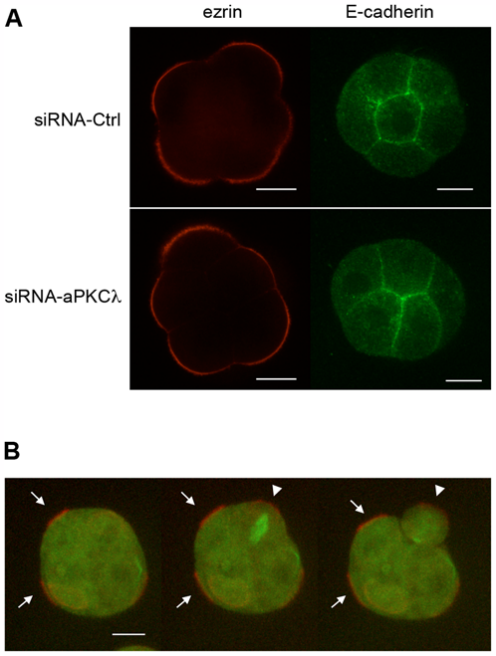
Establishment of apico-basal polarity at the 8-cell stage. (A): Immuno-localization of ezrin (31 embryos) and E-cadherin (23 embryos) during compaction in control siRNA (siRNA-Ctrl) or siRNA-aPKCλ injected embryos. siRNA were microinjected at the 2-cell stage. Scale bars: 20 µm. (B): Spinning-disk video-microscope analysis of a siRNA-aPKCλ injected embryo expressing exogenous tubulin-GFP and ezrin-mCherry during the 8- to 16-cell stage transition. Pictures were taken from the video S3 shown in the supporting information section. mRNA coding for tubulin-GFP, mRNA coding for ezrin-mCherry, and siRNA-aPKCλ were microinjected at the 2-cell stage. Arrows point out apical ezrin accumulation, and arrowheads indicate stable ezrin apical staining during and after mitosis. Scale bar: 20 µm. See the supporting information section for the corresponding videos S2 and S3.

The setting up of cell polarity in mouse preimplantation embryo occurs during compaction at the eight-cell stage. Therefore, we analyzed the localization of ezrin, a protein that is restricted to the apical cortex of outer cells [24] and E-cadherin, the major cell adhesion molecule involved in intercellular flattening in the preimplantation embryo [25], as markers of the apical and basolateral domains, respectively, in compacted 8-cell stage embryos. Moreover, the anti-cadherin antibody used allows the detection of cadherin molecules involved in homophilic interactions. Both markers were correctly localized in the absence of aPKCλ (Fig. 2A), indicating that the setting up of cell polarity along a radial axis was not impaired.

The stability of cortical polarity during mitosis from the 8- to 16-cell stage is essential for the divergence between the inner and outer lineages [3]. Embryos expressing both tubulin-GFP and ezrin-mCherry fusion proteins were analyzed during the 8- to 16-cell stage transition by time-lapse video-microscopy (Fig. 2 and videos S2 and S3). This approach confirmed that ezrin was accumulated at the apical pole during compaction (Fig. 2B, arrows) and remained stable during mitosis (Fig. 2B, arrowheads).

These results indicate that aPKCλ is not required for the setting up of cell polarity at the 8-cell stage nor for its stability during mitosis, at the 8- to 16-cell stage transition.

#### aPKCλ depletion causes a polarity defect at the 16-cell stage

**Figure 3:**
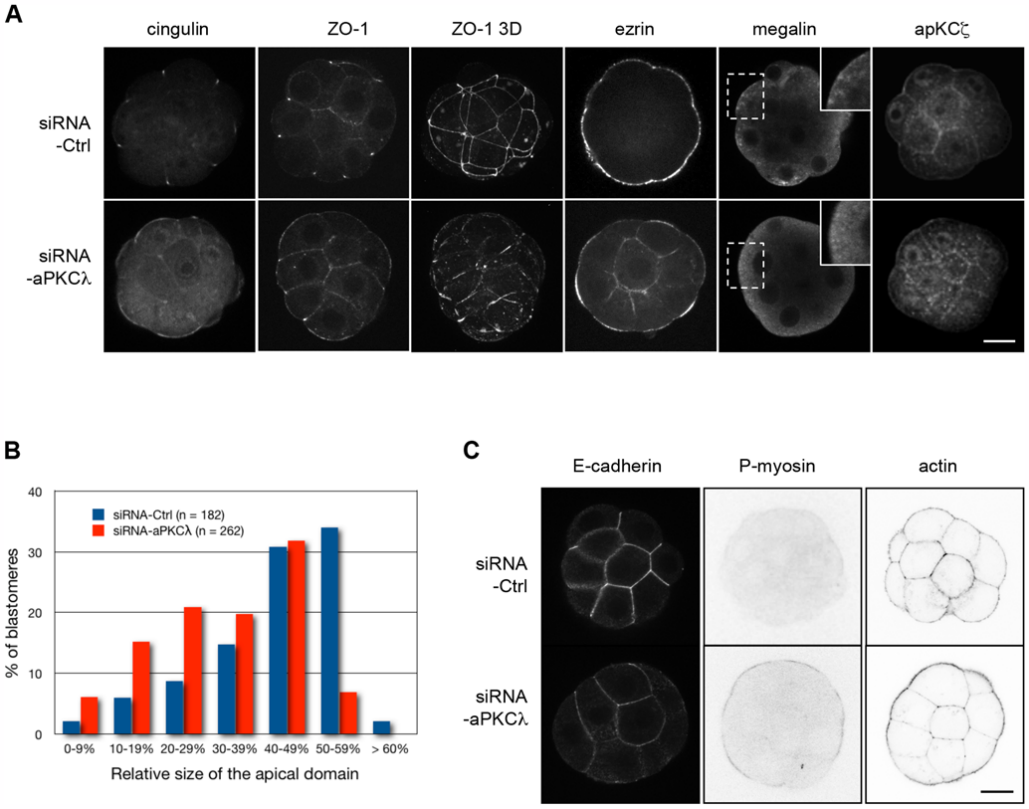
Abnormal cell polarity at the 16-cell stage in absence of aPKCλ expression. (A): Confocal microscope analysis of cingulin (27 embryos), ZO1 (34 embryos), ezrin (39 embryos), megalin (9 embryos), and aPKCζ (28 embryos) in control siRNA (siRNA-Ctrl) or siRNA-aPKCλ injected embryos. Figures ZO1-3D represent projections of 30 confocal sections to visualize the belt-like staining of ZO1. siRNA-Ctrl or siRNA-aPKCλ were microinjected at the 2-cell stage. Scale bar: 20 µm. (B): Measure of the apical surface in control (blue bars) and in aPKCλ-depleted (red bars) blastomeres. The measure represents the percentage of the apical membrane relative to the whole plasma membrane. The difference between the two groups is highly significant (p<0.0001). (C): Confocal microscope analysis of E-cadherin (32 embryos), phospho-myosin (37 embryos), and actin (49 embryos), at the 16-cell stage in control-siRNA (siRNA-Ctrl) or siRNA-aPKCλ injected embryos. siRNA-Ctrl or siRNA-aPKCλ were microinjected at the 2-cell stage. Scale bar: 20 µm.

**Figure 4:**
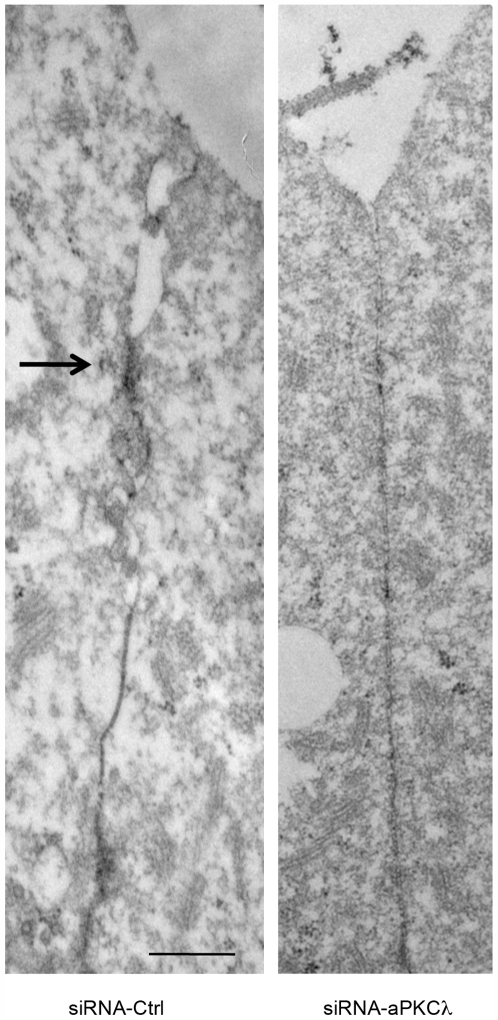
Tight junction formation is impaired in aPKCλ-depleted embryos. Electron microscope analysis of 16-cell stage control siRNA (siRNA-Crtl) or siRNA-aPKCλ injected embryos. Arrow points at tight junction. Scale bar: 0.5 µm.

From the 16-cell stage up to the mid 32-cell stage, outer cells progressively differentiate into epithelial cells. One of the major events is the maturation of junctions, characterized by the step-by-step accumulation of tight junction components at the border between the apical and basolateral domains [26]. Tight junctions form a diffusion barrier between these two membrane domains and thus stabilize cell polarity. In control embryos, ZO-1 and cingulin, two components of the tight junctions, were detected as apico-lateral dots between outer cells forming a characteristic belt-like staining in 3D reconstructions (Fig. 3A) as previously described [27], [28]. In siRNA-aPKCλ injected embryos, ZO-1 and cingulin were mislocalized to the apical pole and diffusely distributed along cell contacts and in the cytoplasm (Fig. 3A). These data suggest that aPKCλ-defective cells show defects in the development of tight junctions. This was confirmed by electron microscopy analysis at the 16-cell stage since we never observed a tight junction structure in siRNA-aPKCλ injected embryos (Fig. 4). Similarly, inactivation of aPKCλ in other systems led to abnormal localization of several tight junction proteins: occludin was localized to the apical membrane in Xenopus embryo [29] and ZO-1, occludin and claudin-1 disappeared from cell-cell boundaries in mammalian epithelial cells [30], [31]. Disruption of tight junctions induces the loss of apico-basal boundaries, which is likely to result in inter-domain diffusion of molecules and impaired asymmetric distribution of membrane proteins. Therefore, the localization of the apical marker ezrin was studied. In control embryos, ezrin was exclusively found at the apical domain of outer blastomeres, whereas some staining was also detected at the basolateral domain, in addition to the apical staining, in aPKCλ-depleted embryos (Fig. 3A). Measurement of the apical and basolateral domains length of blastomeres revealed that inactivation of aPKCλ resulted in the restriction of the apical domain (Fig. 3B) as observed in Drosophila, mouse and Xenopus epithelia [29], [32]. Finally, while the endocytic receptor megalin was observed at the apical membrane and displayed a punctuate distribution in the apical cytoplasmic domain of control embryos, it was absent from the apical membrane and diffusely distributed in the cytoplasm in aPKCλ-depleted embryos (Fig. 3A), suggesting that cytoplasmic polarity was also impaired. This is consistent with previous studies reporting a role for PAR proteins in endocytosis [33], [34]. The correct localization of aPKCζ at the apical domain in absence of aPKCλ (Fig. 3A) indicates that aPKC isoforms display specific functions during preimplantation development as observed in other systems [23], [35].

Since aPKCλ depleted embryos displayed a very compacted aspect at the 16-cell stage (Fig. 1D), the distribution of E-cadherin was investigated. Surprisingly, staining of E-cadherin engaged in homophilic interactions was weaker in aPKCλ-depleted embryos (Fig. 3C), although we did not observe any significant redistribution of E-cadherin into the cytosol and expression of total E-cadherin was not modified (data not shown). By contrast, we observed a strong accumulation of phospho-myosin at the apical cortex in aPKCλ-depleted embryos compared to control embryos (Fig. 3C), indicating that apical myosin II is up-regulated. This up-regulation is generally correlated to a higher cortical tension [36]. By comparison with other systems, these results suggest that tissue apical cortical tension is higher in those embryos. Indeed, the modification of aPKC expression in in vitro cultured epithelial explants leads to the formation of compact aggregates that minimize their surface [37]. Therefore, the depletion of aPKCλ leads embryos to minimize their apical surface area [37], [38] and acquire a ball-like shape. Adhesion and cortical tension are inter-dependent because both are supported by actin filaments. It has been described that a decrease in the number of cortical microfilaments stabilizing E-cadherin complexes and an increase in the number of microfilaments anchored to the plasma membrane through ezrin would increase cortical tension [38]–[42]. Interestingly, we have observed a reorganization of actin cytoskeleton characterized by an increase of actin staining at the apical cortex at the expense of cell-cell contacts area in aPKCλ-depleted embryos (Fig. 3C). This observation may explain the reduction of E-cadherin staining. Taken together, our results suggest that the ball-like shape observed in aPKCλ-depleted embryos is rather due to a higher cortical tension than to a stronger cell-cell adhesion.

### Cell fate depends upon the cellular context when aPKCλ function is lost

It is at the 16-cell stage that progenitors of inner cell mass (inside non-polarized cells) and trophectoderm (outside polarized cells) emerge. Therefore, we analyzed the influence of aPKCλ down-regulation on the divergence between these two lineages. To investigate the role of the cellular context on these events, we inactivated aPKCλ in whole or half embryos. In order to inactivate aPKCλ in all blastomeres, we have microinjected siRNAs and/or mRNAs encoding aPKCλ kinase dead (aPKCλ-K273E). However, since siRNA may pass through mid-bodies that connect sister cells and persist during two cell cycles in the embryo [43], we microinjected mRNA coding for aPKCλ-K273E fused to GFP to interfere with the function of aPKCλ only in half blastomeres. Moreover, this construct allowed us to follow the lineage of the injected blastomere because of the GFP.

#### Strong reduction of the number of inside 16-cell stage blastomeres when aPKCλ is depleted in all cells

**Figure 5:**
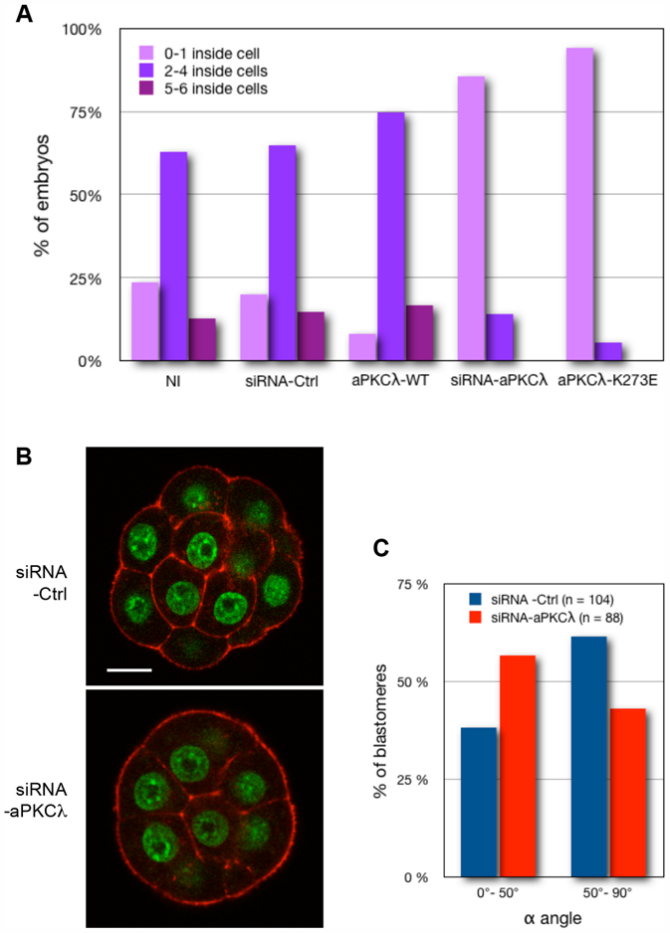
aPKCλ-depletion leads to a strong reduction of inner cell number. (A): The number of inner cells per embryo at the 16-cell stage was determined in non-injected control embryos (NI), siRNA-aPKCλ, or control siRNA (Ctrl-siRNA) injected embryos, in aPKCλ-K273E or aPKCλ-WT expressing embryos. siRNA or mRNA were microinjected at the 2-cell stage. (B): Embryos were recovered at the 16-cell stage and stained for actin and chromatin (red and green staining respectively). Whole embryos were scanned along the z-axis using a confocal microscope to count inner and outer cells. Cells were considered as outer or inner on the basis they had or not a contact-free membrane. Stacks of images of injected embryos are available in the supporting information section (videos S4 and S5). (C): Analysis of spindle orientation during the 8- to 16-cell stage transition. Distribution of α angle value in blastomeres from control-siRNA (blue bars) or siRNA-aPKCλ (red bars) injected embryos undergoing the 8- to 16-cell stage transition. Asymmetric divisions take place when α angle is lower than 50°. The difference between the two groups is significant (p = 0,013; Fisher's exact test).

Embryos were stained for actin and chromatin, and optically sectioned to visualize cell shape and count the number of inner and outer cells (stacks of confocal acquisitions are available in the supporting information section, videos S4 and S5). Non-injected control embryos (n = 46), siRNA control embryos (n = 20), and aPKCλ-WT expressing embryos (n = 12) displayed a broad range of inner cells number at the 16-cell stage, going from 0 to 6 inner cells per embryo, corresponding to a mean value of about 3 inner cells per embryo (Table 2 and Fig. 5). In aPKCλ depleted embryos (n = 21) and aPKCλ-K273E expressing embryos (n = 18), the number of inner cells per embryo was narrowed down between 0 and 2 inner cells, with a mean value of 0.8 inner cells (Table 2 and Fig. 5).

**Table 2:** Effect of aPKCλ-depletion on cell allocation in 16- and 32-cell stage embryos.

Microinjected RNA (in both 2-cell stage blastomeres)	Number of embryos analyzed	Number of inner cells (mean**±**SEM)	Number of outer cells (mean**±**SEM)
16-cell stage			
siRNA-aPKCλ	21	0.8±0.2*	15.4±0.2
siRNA-Crtl	20	3.1±0.4	13.1±0.3
mRNA aPKCλ-K273E	18	0.8±0.2**	15.3±0.2
mRNA aPKCλ-WT	12	3.2±0.5	13.0±0.5
Non-injected Ctrl	46	2.6±0.2	13.5±0.2
32-cell stage			
siRNA-aPKCλ	11	9.2±0.8	22.4±0.7
siRNA-Ctrl	5	9.0±0.8	20.8±1.0
mRNA aPKCλ-K273E	9	7.4±0.9***	23.6±0.9
mRNA aPKCλ-WT	16	10.6±0.6	21.0±0.6
Non-injected Ctrl	42	10.7±0.4	20.6±0.4

*p<0.0001 using the unpaired t-test with Welch correction when siRNA-aPKCλ samples were compared to siRNA-Ctrl and non-injected Ctrl respectively.

**p<0.0001 using the unpaired t-test with Welch correction when mRNA aPKCλ-K273E samples were compared to mRNA aPKCλ-WT and non-injected Ctrl respectively.

***p<0.012 using the unpaired t-test with Welch correction when mRNA aPKCλ-K273E samples were compared to mRNA aPKCλ-WT.

The differences between siRNA-aPKCλ samples and siRNA-Ctrl and non-injected Ctrl respectively were not significant (using the unpaired t-test with Welch correction).The differences between the siRNA-Ctrl, mRNA aPKCλ-WT and non-injected Ctrl were not significant using the unpaired t-test with Welch correction.

When embryos were recovered and analyzed at the 32-cell stage, the number of inner cells was not significantly different between mutant and control embryos, although embryos expressing aPKCλ-K273E contained a slightly lower number of inner cells (Table 2). This demonstrates that aPKCλ-depleted blastomeres can contribute to both lineages to the same extent as wild-type blastomeres.

#### aPKCλ down regulation favors asymmetric divisions during the 8- to 16-cell transition

The decrease of inner cells in aPKCλ-depleted embryos may be explained by an increase in the number of symmetric divisions during the 8- to 16-cell stage transition. In order to analyze the orientation of mitotic spindles during this transition, 3D movies of embryos expressing a tubulin-GFP fusion protein were recorded by video-microscopy. From these movies, we measured the α angle formed between two vectors: the first vector defined by the two poles of the metaphase spindle of each dividing blastomere, and the second one corresponding to the radial axis going from the centroid of the embryo to the center of each mitotic spindle (corresponding to the apico-basal axis of polarity of blastomeres). Once the spindle formed, its orientation did not change until telophase. From our data, asymmetric divisions take place when the α angle formed by the spindle axis and the radial axis (axis of polarity of the cell) is lower than 50° (we observed a mean number of 3.1 inside cells/embryo (see Table 2) corresponding to 39% of asymmetric divisions and the threshold α angle value was at 50° for 39% (Fig. 5C, blue bars)). In absence of aPKCλ expression, the orientation of spindles differs when compared to control embryos (Fig. 5C, red bars): this α angle was below 50° in 57% of the aPKCλ-depleted embryos (compared to 39% in control embryos). These results indicate that upon inhibition of aPKCλ expression a higher proportion of mitotic spindles tend to align with the axis of polarity. These data also reveal that cell allocation within the embryo does not systematically reflect the orientation of spindle during mitosis. Therefore, attempts to conclude on symmetric versus asymmetric divisions from the analysis of cell position at the 16-cell stage may lead to errors of interpretation if spindle orientation is not checked in parallel [16], [19].

**Figure 6:**
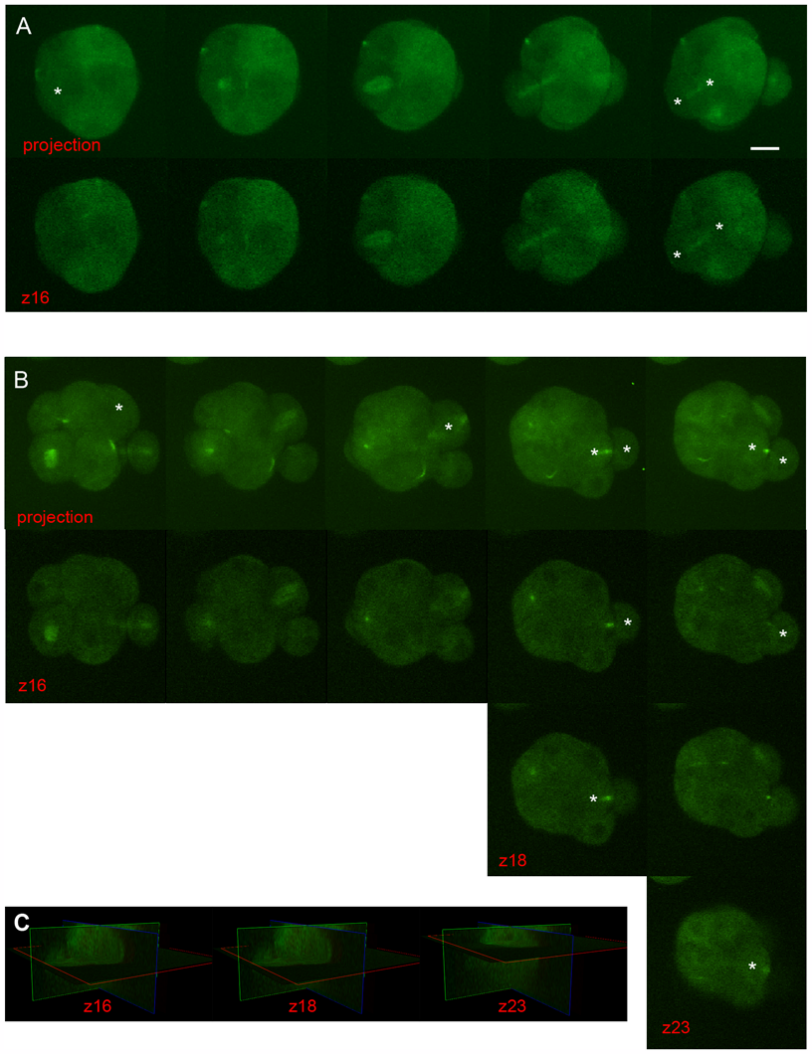
Relocation of blastomeres at the periphery after an asymmetric division in aPKCλ depleted embryos. (A): Spinning disk microscope analysis of a siRNA-Crtl injected embryo expressing exogenous tubulin-GFP during the 8- to 16-cell stage mitosis (time between images: 20 min). Upper panel: projection of 30 Z planes (Z step, 2 mm). Bottom panel: only the 16th z plane is shown (corresponding to the center of the embryo. The asterisks show the mother cell and the two daughter cells after an asymmetric division. Note that the two daughter cells are in the same plane and that the inside cell is correctly localized. siRNA were microinjected at the 2-cell stage. Scale bar: 20 µm. (B): Spinning disk microscope analysis of a siRNA-aPKCλ injected embryo expressing exogenous tubulin-GFP during the 8- to 16-cell stage mitosis. Upper panel: projection of 31 Z planes (Z step, 2 mm). Bottom panels: the 16th, 18th and 23rd z planes are shown. Note that the “inside” daughter cell moves toward the top and periphery of the embryo (from the 16th z planes to the 18th 20 min. later and finally to the 23rd z plane). siRNA were microinjected at the 2-cell stage. (C): 3D images showing the position of the three different z planes (in red).

Since cortical polarity is stable during mitosis in aPKCλ-depleted embryos during the 8-cell to 16-cell stage transition, this preferential spindle orientation should generate a higher number of inside cells at the 16-cell stage (from 3 to 4). However, a decrease in this number was observed (from 3 to 0.8). A more careful analysis of movies suggests that rapidly after mitosis, the cell that inherits the basolateral side of the mother cell (when the division is asymmetric) does not display an inner allocation but rather relocates at the periphery of the embryo and starts to polarize (due to the asymmetry of contacts) (Fig. 6). This reorganization could be achieved because cortical polarity is not stabilized in aPKCλ-depleted blastomeres (allowing flexibility of the different cortical domains in polarized cells) and because of a stronger cortical tension. The relative tissue surface tension for both cell types would be similar, making differential adhesion less effective, as suggested by the decrease in E-cadherin staining (Fig. 3C). Thus, inner cells would tend to keep a more rounded shape and outer polarized cells would not tend to engulf inside cells, thereby leading non polarized cells to move to the periphery, where they will develop an apical pole of microvilli, due to the asymmetry in cell contacts [44].

#### The effect of aPKCλ down regulation is dependent upon the cellular context

In most multi-cellular organisms, the stabilization of a polarized phenotype is dependent upon cell-cell interactions. In order to study the behavior of aPKCλ depleted blastomeres in a different cellular context, we decided to study the effect of aPKCλ inactivation in only part of the embryo. mRNAs encoding either wild-type aPKCλ (aPKCλ-WT) or kinase dead aPKCλ (aPKCλ-K273E) fused to GFP were injected in one blastomere at the two-cell stage. The mean proportion of GFP-tagged blastomeres was similar to untagged blastomeres in both groups of embryos at the 16- and 32-cell stages (Table 3), indicating that expression of these aPKCλ constructs had no effect on cell division rate. We did not notice any significant difference at the 16-cell stage, aPKCλ-WT and aPKCλ-K273E expressing blastomeres contributing to inner and outer cells to the same extent (Table 3). By contrast, at the 32-cell stage, the contribution of aPKCλ defective blastomeres to inner cell mass was significantly higher compared to blastomeres derived from the non-injected blastomere (Table 3). Indeed, 10 out of 16 (≈62%) aPKCλ-K273E expressing cells were found in the inner cell mass, representing almost 3/4 of the total inner cell mass population. By contrast, 5 out of 16 aPKCλ-WT expressing cells were detected in the inner cell mass and represented about 45% of the total inner cell mass population. Consistent with these results, the number of inner cells was higher in aPKCλ-K273E injected embryos (14.0; 42%) compared to aPKCλ-WT (11.4; 36%) injected ones or non-injected controls (10.7; 34%). Thus, the mosaic disruption of aPKCλ activity in embryos interferes with the ability of blastomeres to contribute to trophectoderm lineage, and biases the progeny of aPKCλ defective blastomeres towards the inner cell mass, in agreement with a previous study [19]. These results also show that almost all outer 16-cell blastomeres expressing aPKCλ-K273E divide asymmetrically while wild-type blastomeres divide symmetrically. This can be also explained by the difference in cortical tension existing between the two cell types: dividing wild-type outer blastomeres will be more flattened than dividing mutant outer blastomeres that exhibit a higher cortical tension.

**Table 3:** Effect of mosaic aPKCλ-inactivation on cell allocation at 16-cell stage.

Microinjected mRNA (in one 2-cell stage blastomere)	Percentage of GFP positive cells (mean**±**SEM)	Number of inner cells (mean**±**SEM)	Number of GFP inner cells (mean**±**SEM)	Number of outer cells (mean**±**SEM)	Number of GFP outer cells (mean±SEM)
16-cell stage					
aPKCλ-K273E (n = 16)	50.4±0.4%	2±0.2*	1±0.2	14±0.2	7±0.2
aPKCλ-WT (n = 14)	50.6±0.6%	3.1±0.3	1.3±0.3	13±0.3	6.8±0.3
Non-injected Ctrl (n = 46)	–	2.6±0.2	–	13.5±0.2	–
32-cell stage					
aPKCλ-K273E (n = 16)	49.9±0.5%	14±0.4^#^	10±0.8##	19±0.3	6±0.8
aPKCλ-WT (n = 14)	48.3±0.9%	11.4±0.4	4.9±0.4	19.4±0.4	9.9±0.3
Non-injected Ctrl (n = 42)	–	10.7±0.4	–	20.6±0.4	–

n: number of embryos analyzed.

*p<0.005 and <0.04 using the unpaired t-test with Welch correction when mRNA aPKCλ-K273E samples were compared to mRNA aPKCλ-WT or non-injected samples respectively.

## p<0.0001 using the unpaired t-test with Welch correction when mRNA aPKCλ-K273E samples were compared to mRNA aPKCλ-WT.

Taken together, these data indicate that disruption of aPKCλ activity results in different phenotypes depending on the cell context. Indeed, we observed a strong drop of inner cell number at the 16-cell stage when aPKCλ activity was inhibited in all blastomeres but not when inhibition was performed in only half of the blastomeres. This difference may result from the normal E-cadherin staining observed at the interface between mutant and normal cells (data not shown), which might block the outward migration of inner cells after the 8- to 16-cell stage transition. Moreover, aPKCλ defective embryos displayed a quasi-normal number of inner cells at the 32-cell stage, suggesting that a compensatory mechanism is at work during the 16- to 32-cell stage transition to re-establish a correct number of inner cells at the blastocyst stage (see Table 2). When aPKCλ activity is suppressed in only half of the blastomeres, this results in an enrichment of aPKCλ defective blastomeres in the inner cell mass at the 32-cell stage. Therefore, these experiments show that the position of aPKCλ-depleted blastomeres is dependent upon both intrinsic and extrinsic factors. Among the intrinsic factors, forces involved in the control of cell shape might be at work when cell polarity is not stabilized.

#### Cell shape control lineage allocation

**Figure 7:**
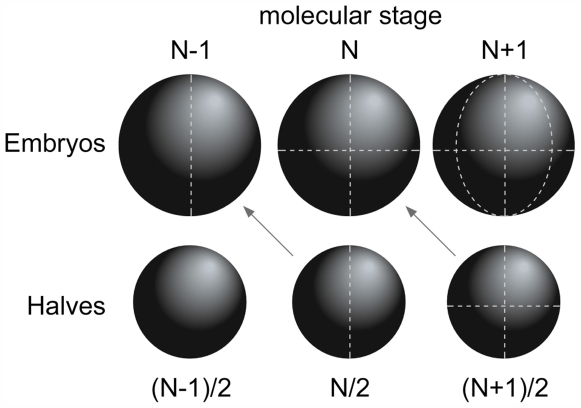
The spatial organization of cells in embryo halves corresponds to the one found in embryos of the preceding molecular stage. A half embryo at the molecular stage N (embryo N/2) will have a cellular 3D organization similar to the one observed in an embryo at the molecular stage N-1.

As stated in the introduction, the role of cell shape in spindle orientation and compensation of the number of inner cells was already known. However, our studies pointed to a role of cell shape in controlling cell position and thus lineage allocation. In our previous experiments, the inactivation of aPKCλ led to changes in cortical tension and adhesive properties. Thus, we decided to test the role of cell shape on cell allocation. We used embryos derived from a single 2-cell blastomere. The only difference between these embryos (called N/2-cell stage) and control embryos (called N-cell stage) at the same molecular stage is that the 3D shape of the N/2-cell stage embryo will be similar (although smaller, but homothetic) to the 3D shape of an N-cell stage embryo at one embryonic stage earlier (Fig. 7): i.e. 32/2-cell stage embryos will be similar to 16-cell stage embryos as far as cell shape is concerned. The percentage of inside cells in 32/2-cell stage embryos was 15% (n = 9) a value similar to the one observed in 16-cell stage embryos (16%; n = 46) and much lower than the one observed in 32-cell stage embryos (41%; n = 42) (Table 4). Since blastomeres in 32-cell stage embryos and in 32/2-cell embryos are exactly at the same molecular age, this result reinforces the conclusion that cell shape plays an important role in lineage allocation. Although the reduction of the number of ICM cell in half-embryos has been previously reported [45] our current data indicate that cell shape is involved in the biased allocation of blastomeres observed in these half-embryos.

**Table 4:** Cell allocation in embryo halves.

	Percentage of inner cells (n = number of embryos)
	16-cell stage	32/2-cell stage	32-cell stage
Non-injected control	16% (n = 30)	15% (n = 9)	32% (n = 21)
siRNA-aPKCλ	5% (n = 21)	14% (n = 5)	29% (n = 11)

In addition these experiments suggest also that a compensatory mechanism is at work at the 32-cell stage in aPKCλ-depleted blastomeres: the percentage of inside cells in 32/2-cell stage aPKCλ-depleted embryos was 14% instead of 5% in aPKCλ-depleted 16-cell embryos. This value is similar to the one observed in 16-cell stage wild-type embryos (16%). This is likely due to the ability of aPKCλ-depleted 32-cell stage blastomeres to establish tight junctions and to stabilize cortical polarity, thus behaving like wild-type cells.

### Conclusion

**Figure 8:**
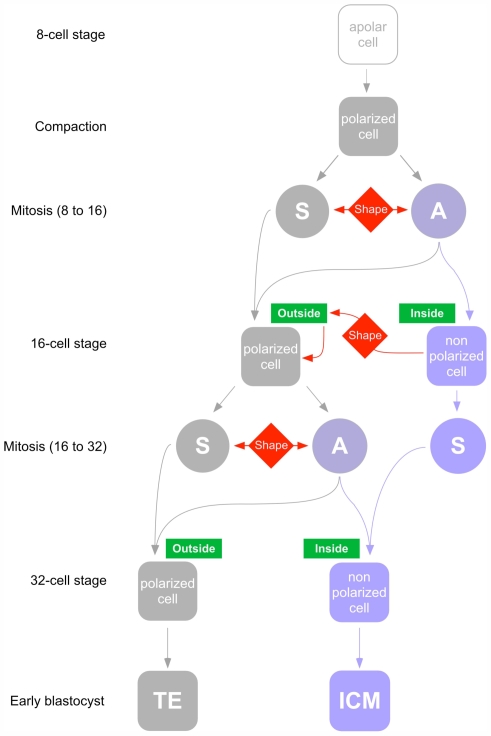
Lineage allocation during mouse preimplantation development. Until the early 8-cell stage, blastomeres are apolar. At compaction, blastomeres polarize and flatten upon each other. During the following mitosis, they divide either symmetrically (S) or asymmetrically (A). Although spindle orientation is random, the ratio between symmetrical and asymmetrical cell divisions can be modulated by cell shape constraints. After division, non-polar cells are localized inside the embryo (since they are derived from the basal part of 8-cell blastomeres) while polar cells are located at the periphery. At the 16-cell stage, before the formation of tight junctions (or in their absence) cell shape constraints can force inside cells to move at the periphery (red arrow). These cells will polarize because of the asymmetry of intercellular contacts. At the 16- to 32- cell transition, in 16-cell stage embryos containing only a small number of inside non-polarized blastomeres, cell shape constraints favor asymmetrical cell divisions in outside polarized blastomeres to increase the number of inside non-polarized cells.

The divergence of lineages between the trophectoderm and the inner cell mass in the preimplantation mouse embryo is dependent upon both intrinsic and extrinsic factors. Our study shows that in addition to cell polarization at compaction and the existence of asymmetric divisions during the following mitosis, the aPKCλ-dependent stabilization of cortical domains during the 16-cell stage is critical for cell lineage divergence. As observed in other systems, aPKCλ plays a major role in the very first steps of tight junctions formation, which are essential to maintain a physical border between the apical and basolateral domains, stabilizing cell polarity and blocking the diffusion of membrane molecules between the apical and basolateral domains of the epithelial cells. Finally, we bring to light for the first time a role for cell shape in interphasic blastomere (dependent upon cortical tension and cell adhesion) in cell allocation: aPKCλ influences cortical tension and intercellular adhesion, thereby controlling cell shape and cell position within the embryo (Fig. 8). Positional information controls cell fate through the asymmetry of cell contact, a major cue for epithelial differentiation. Cell shape also modulates the ratio between symmetric and asymmetric divisions at the 8- to 16- and 16- to 32-cell stage, thus controlling the number of cells allocated to each of the first two lineages. The interplay between these different factors explains the robustness and the highly regulative aspects of preimplantation development. It is only when a blastocyst is formed, with its cavity and the two established cell lineages, that cell fate is fixed through the differential expression of tissue-specific transcription factors allocation [14], [15].

## Supporting Information

Video S1 Video-microscope analysis of control-siRNA (left panel) and siRNA-aPKCλ (right panel) injected embryos. Two-cell stage embryos were microinjected with siRNA (siRNA-aPKCλ or siRNA-Ctrl) in both blastomeres and visualized under a spinning-disk videomicroscope during the 8- and 16-cell stages. Acquisitions were performed every 30 minutes.
(2.52 MB MOV)

Video S2 Video-microscope analysis of control-siRNA injected embryos expressing tubulin-GFP and ezrin-mCherry. Two-cell stage embryos were microinjected with siRNA-Ctrl mixed with mRNA (tubulin-GFP mRNA and ezrin-mCherry mRNA) in both blastomeres and visualized under a spinning-disk videomicroscope during the 8- to 16-cell stage transition. Acquisitions were performed every 30 minutes.
(0.77 MB MOV)

Video S3 Video-microscope analysis of siRNA-aPKCλ injected embryos expressing tubulin-GFP and ezrin-mCherry. Two-cell stage embryos were microinjected with siRNA-aPKCλ mixed with mRNA (tubulin-GFP mRNA and ezrin-mCherry mRNA) in both blastomeres and visualized under a spinning-disk videomicroscope during the 8- to 16-cell stage transition. Acquisitions were performed every 30 minutes.
(1.84 MB MOV)

Video S4 Stack of confocal sections of control siRNA injected embryos stained for actin (red) and chromatin (green).
(10.23 MB MOV)

Video S5 Stack of confocal sections of siRNA-aPKCλ injected embryos stained for actin (red) and chromatin (green).
(8.65 MB MOV)
